# Delayed Diagnosis of Myxedema Coma in a Patient With Concurrent Severe Intracranial Atherosclerotic Disease

**DOI:** 10.7759/cureus.83339

**Published:** 2025-05-02

**Authors:** Adam Tolaymat, George Mitchell

**Affiliations:** 1 Medical School, VCOM (Edward Via Virginia College of Osteopathic Medicine) - Carolinas, Spartanburg, USA; 2 Critical Care Medicine, Cleveland Clinic Indian River Hospital, Vero Beach, USA

**Keywords:** cerebro-vascular accident (stroke), hypothermia, hypothyroid myxedema coma, hypothyroid pericardial effusion, metabolic encephalopathy

## Abstract

Myxedema coma is a severe and life-threatening presentation of hypothyroidism that is less prevalent in more developed countries. It is associated with hypothermia, altered mental status, and multi-system organ failure and can be precipitated by acute stressors like infection, surgery, or myocardial infarction. Rapid diagnosis and treatment of myxedema coma are imperative due to the significant mortality seen. In this case, an 83-year-old male presented to the emergency department intubated after being found unresponsive on the floor of his home by family members. Initial examination with labs and imaging led to suspicion of acute hypoxic respiratory failure and acute metabolic encephalopathy, potentially due to an ischemic event. Imaging confirmed multiple cerebellar infarcts and severe intracranial atherosclerotic disease. The absence of clinical improvement led to further laboratory and physical examination workup, which resulted in a delayed diagnosis of myxedema coma precipitated by ischemic brain injury. Once the diagnosis was confirmed, treatment was initiated with thyroid hormone replacement and glucocorticoid supplementation. Slight initial improvement was seen after treatment with incremental rises in free thyroxine hormone levels and decreases in thyroid-stimulating hormone (TSH), but the patient’s medical power of attorney decided on comfort measures only, and the patient ultimately expired. This report should highlight the importance of early suspicion and diagnosis of myxedema coma in patients who present with altered mental status, bradycardia, hypothermia, and anemia in the setting of other potential confounding diagnoses.

## Introduction

Myxedema coma is an uncommon endocrine emergency that may present due to untreated or undiagnosed severe hypothyroidism and is associated with high mortality due to its rarity and insufficient clinical data [1]. Incidence rates of myxedema coma have been reported as 0.22 cases per million per year [2], though rates may be as high as 1.08 cases per million per year, as seen in Japan [3]. The main features of myxedema coma include altered mental status, hypothermia, anemia, and hyponatremia [1], but patients may present with missing certain key features or may present with other symptoms, including weight gain, seizures, and bradycardia [4]. It may manifest itself after acute stress events like pneumonia [5], surgery [6], or IgA vasculitis [7] in patients with untreated hypothyroidism. If left untreated, myxedema coma may progress to respiratory and circulatory failure [2]. Prompt diagnosis and treatment of the condition are required for a good prognosis [3].

Prompt treatment for myxedema coma should begin before a laboratory confirmation of the disease due to the severity of the condition. Standard treatment for myxedema coma involves supportive measures, replacement of thyroid hormone with levothyroxine and liothyronine [8], and glucocorticoid administration until adrenal insufficiency can be excluded. Patients also require close monitoring in an intensive care unit due to increased risks of arrhythmias, myocardial infarction, and unstable sodium levels once beginning treatment [9], as well as the potential complications of the disease. Diagnosis is confirmed through laboratory thyroid function testing with elevated thyroid-stimulating hormone (TSH) levels and decreased levels of free T4. Even with prompt diagnosis and treatment, mortality rates remain high, with studies showing mortality rates above 50% [10,11].

## Case presentation

An 83-year-old Caucasian male with a past medical history of atrial fibrillation and a history of stroke presented to the emergency department with altered mental status and had been intubated by the emergency medical services personnel after being found unresponsive at home hanging off his bed by family members. He presented in the late afternoon after last being seen well earlier in the morning by his family. He presented hypotensive with a blood pressure of 97/60 mmHg, a pulse of 131, oxygen saturation of 98%, and a temperature of 36.3 °C. He was unresponsive to verbal or painful stimuli and the Glasgow Coma Scale scoring was 3. His mucous membranes were dry, and his skin was pale. Anisocoria was present with the right pupil dilated to 8 mm and the left pupil at 2 mm, with both pupils nonreactive to light and accommodation.

Initial laboratory testing disclosed acute kidney injury with an elevation in blood urea nitrogen (BUN) and creatinine to 26 mg/dL and 1.30 mg/dL, respectively, with a decrease in glomerular filtration rate (GFR) to 55 mL/min/1.73 m^2^. Troponin level was 93 ng/L. Lactate was elevated at 4.5 mmol/L, but arterial blood gas was consistent with a respiratory alkalosis with a pH of 7.59, arterial pCO_2_ (partial pressure of carbon dioxide) of 23 mmHg, and a decreased pO_2 _(partial pressure of oxygen)/FiO_2 _(fraction of inspired oxygen) ratio of 183. Lab findings are summarized in Table 1, with blood gas findings summarized in Table 2. Chest X-ray noted bilateral basilar atelectasis, and non-contrast CT of the head was negative for acute intracranial hemorrhage or mass effect. Electrocardiogram findings were significant for atrial fibrillation with rapid ventricular response, and a potential septal infarct non-ST-elevation myocardial infarction (NSTEMI) could not be ruled out. He was started on IV hydration, and respiratory viral panels, blood cultures, and sputum cultures were collected before initiating empiric antibiotic treatment with piperacillin-tazobactam 3.375 g IV. He was then admitted to the intensive care unit, where the continued hypotension warranted administration of 10 mcg/min of norepinephrine.

**Table 1:** Laboratory findings on admission BUN, blood urea nitrogen; GFR, glomerular filtration rate; RBC, red blood cells.

Lab test	Patient's value	Normal range
Sodium (mmol/L)	134	136-144
Potassium (mmol/L)	3.9	3.7-5.1
Chloride (mmol/L)	98	98-107
BUN (mg/dL)	26	9-24
Creatinine (mg/dL)	1.30	0.73-1.22
GFR (mL/min/1.73 m^2^)	55	>60
RBC (m/uL)	3.12	4.20-6.00
Hemoglobin (g/dL)	11.6	13-17
Initial high-sensitivity troponin T (ng/L)	93	<12
Second high-sensitivity troponin T (ng/L)	108	<12
Third high-sensitivity troponin T (ng/L)	195	<12
Lactic acid (mmol/L)	4.5	0.5-2.2
Creatine kinase (U/L)	173	51-298

**Table 2:** Arterial blood gas findings on admission pCO_2_, partial pressure of carbon dioxide; pO_2_, partial pressure of oxygen; FiO_2_, fraction of inspired oxygen.

Lab test	Patient's value	Normal range
pH, arterial	7.59	7.35-7.45
pCO_2_, arterial (mmHg)	23	36-46
pO_2_, arterial (mmHg)	183	85-95
Bicarbonate, arterial (mmol/L)	22	22-26
pO_2_/FiO_2_ ratio	183	>300

**Figure 1:**
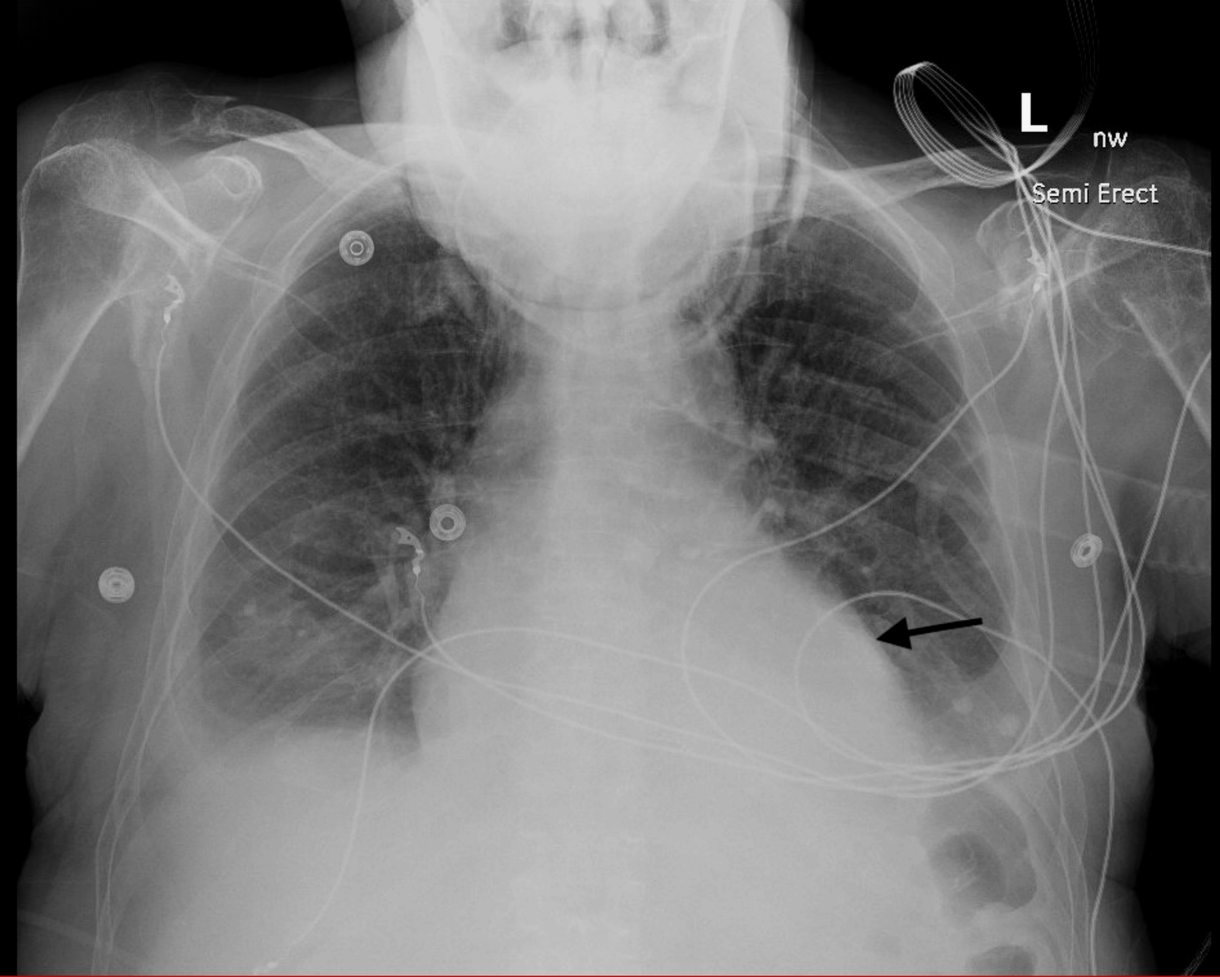
Semi-erect chest X-ray showed pericardial effusion.

A cardiology workup involved a chest X-ray which noted a large pericardial effusion as seen in Figure 1.

Neurology workup involved an electroencephalogram, which indicated metabolic encephalopathy, but no epileptic activity was noted. CT angiography noted multiple intracranial points of stenosis in the left middle cerebral, right anterior cerebral, and right posterior cerebral arteries with severe intracranial atherosclerotic disease. He remained encephalopathic and lethargic but was able to open his eyes to noxious stimuli. Continued fluid resuscitation resulted in an improvement in kidney function as BUN and creatinine returned to near-baseline values of 20 mg/dL and 0.98 mg/dL, respectively. His respiratory cultures returned with evidence of *Klebsiella pneumonia,* and the patient was started on cefepime. His hypotension improved on the third hospital day. Norepinephrine was discontinued on the fifth hospital day. Upon discontinuation of norepinephrine, his blood pressure continued to fluctuate and dropped to 82/52 mmHg. He was started on midodrine 10 mg every 8 hours. This treatment was continued over the course of six days.

The patient’s medical power of attorney declined pericardiocentesis of a large pericardial effusion found on echocardiography. On the sixth hospital day, the patient had limited clinical improvement, with hypothermia at 35.8 °C, lack of response to command or withdrawal to painful stimuli, and failed spontaneous breathing trials in attempts to extubate. With no significant clinical improvement noted, other potential causes of this presentation were evaluated, and thyroid function tests were ordered. Thyroid function tests revealed free T4 levels below laboratory minimum of 0.3 ng/dL and a TSH level of 85.3 mIU/L. These laboratory findings in conjunction with the clinical picture and pericardial effusion confirmed a diagnosis of myxedema coma, and the patient was started on hydrocortisone 100 mg IV every 8 hours and 200 mcg of IV levothyroxine. The hydrocortisone dose was decreased to 50 mg IV every 8 hours after two days and further down to 25 mg IV before the patient was transitioned to methylprednisolone 40 mg daily. He received two doses of 76 mcg levothyroxine IV and 2.5 mcg of liothyronine every 8 hours. After three days of treatment, slight improvements were seen in thyroid function laboratory studies, with free T4 increasing to 0.5 ng/dL and TSH decreasing to 41.5 mIU/L, as shown in Table 3. Clinical improvement was also noted as he was able to spontaneously open his eyes, track movement, and nod his head appropriately to simple questions by the second day of this treatment.

**Table 3:** Progression of thyroid function test results TSH, thyroid-stimulating hormone.

Lab result	TSH (mIU/L)	Free T4 (ng/dL)	Free T3 (pg/mL)
Normal value	0.270-4.200	0.9-1.7	2.3-4.1
Patient's value (hospital day 6)	85.300	<0.3	Not collected
Patient's value (hospital day 7)	96.400	<0.3	Not collected
Patient's value (hospital day 8)	51.800	0.4	Not collected
Patient's value (hospital day 9)	44.900	0.5	Not collected
Patient's value (hospital day 10)	41.500	0.5	<0.4
Patient's value (hospital day 11)	31.800	0.6	0.8
Patient's value (hospital day 13)	19.400	0.6	0.9
Patient's value (hospital day 14)	24.700	0.9	1.0
Patient's value (hospital day 16)	25.100	0.8	1.1

On hospital day 11, he was noted to be unresponsive and hypotensive with a blood pressure of 67/48. Norepinephrine was restarted. He improved hemodynamically. He passed a spontaneous breathing trial and was successfully extubated on hospital day 15. He was transferred to the medical floor, but again declined, and a rapid response was called after he became unresponsive with a blood pressure of 58/34 mmHg. Midodrine was started at 15 mg three times daily via the nasogastric tube. He was unable to manage secretions and frequent periods of apnea resulted in decreasing oxygen saturation to 78% at one point. The patient was placed on high-flow oxygen therapy at 60 L/min. Conversations with the patient’s medical power of attorney resulted in the patient being transferred to hospice care, where he ultimately expired on hospital day 21.

## Discussion

This case features a classic presentation of myxedema coma that wasn’t immediately considered due to underlying cerebrovascular disease. This patient did not have documented hypothyroidism, which may have led to the delay in diagnosis. He was refractory to supportive treatment following the stroke and was initially only slightly responsive to treatment once the diagnosis of myxedema coma was confirmed. There were clinical signs present that suggested a diagnosis of myxedema coma, including hypothermia, hypotension, cardiovascular instability, altered mental status, and pericardial effusion. Myxedema coma was likely to have been precipitated by the acute stroke that the patient suffered, but this event also made it difficult to identify the underlying hypothyroidism that was present. Diagnosis and immediate treatment should begin once myxedema coma is suspected due to the high mortality rates, even before confirmation with thyroid function testing.

Treatment for myxedema coma involves thyroid hormone replacement with both levothyroxine and liothyronine [12], supportive measures to support blood pressure, electrolytes, and body temperature, as well as glucocorticoid replacement until a concurrent adrenal insufficiency can be excluded. A previous study showed that high-dose thyroid hormone replacement, specifically doses larger than 500 mcg of levothyroxine or 75 mcg of liothyronine, was associated with poor outcomes [13]. This patient was started on an appropriate treatment regimen immediately once myxedema coma was suspected, but he was unable to recover and ultimately expired. We find that this case serves as a reminder to clinicians to recognize the signs and symptoms of myxedema coma quickly, even in cases where other diagnoses are present and may be confounding the clinical picture. It is imperative that recognition of disease and initiation of treatment occur quickly in order to limit a poor outcome.

## Conclusions

We present a case of myxedema coma precipitated by ischemic brain injury with comatose presentation and pericardial effusion. With a high mortality rate, it is imperative to consider myxedema coma in a patient presenting with altered mental status, hypothermia, and pericardial effusion regardless of a history of thyroid disease or other comorbidities at presentation. Delayed diagnosis and treatment initiation may lead to clinical deterioration and ultimately death. Clinicians should keep myxedema coma in their differential and should consider thyroid function studies as part of an initial workup in the appropriate clinical setting.
